# Microbiome analysis reveals that *Ralstonia* is responsible for decreased renal function in patients with ulcerative colitis

**DOI:** 10.1002/ctm2.322

**Published:** 2021-03-04

**Authors:** Jung Min Kim, John Hoon Rim, Da Hye Kim, Hye‐Youn Kim, Sung Kyoung Choi, Dong Yun Kim, Yo Jun Choi, Seyoung Yu, Jae Hee Cheon, Heon Yung Gee

**Affiliations:** ^1^ Department of Pharmacology Graduate School of Medical Science Brain Korea 21 Project Yonsei University College of Medicine Seoul Republic of Korea; ^2^ Department of Internal Medicine and Institute of Gastroenterology Yonsei University College of Medicine Seoul Republic of Korea; ^3^ Severance Biomedical Science Institute Yonsei University College of Medicine Seoul Republic of Korea; ^4^ Department of Medicine Physician‐Scientist Program Yonsei University Graduate School of Medicine Seoul Republic of Korea; ^5^ Department of Applied Mathematics Hanyang University (ERICA) Ansan Republic of Korea

Dear Editor,

The causal link between inflammatory bowel disease (IBD) and chronic kidney disease (CKD) is not clear; therefore, we aimed to investigate the role of gut microbiota in decreasing renal function in patients with IBD. IBD is characterized by the disruption of host–microbe relationships, and dysbiosis and the metabolites produced by the dysbiotic intestinal microbiome may negatively influence the renal function.^1^ Indeed, epidemiological studies have shown that the prevalence of CKD is higher in individuals with ulcerative colitis (UC) compared to those without UC.^2^, ^3^

To reveal the connection between UC and CKD, we performed 16S ribosomal DNA sequencing using ileocecal mucosal samples from nine patients with both UC and CKD, 29 UC patients with normal renal function, and 12 healthy individuals who had normal colonoscopy results (Table S1; Figure S1). The patients with CKD showed estimated glomerular filtration rate (eGFR) values less than 60 mL/min/1.73 m^2^ for a minimum of 3 months.

The bacterial operational taxonomic units of patients in the UC + CKD group were decreased but were not significantly different from those of the UC or control group (Figure S2A>). Community richness, such as the Chao1 index and abundance‐based coverage estimator (ACE), was not different among the three groups (Figure S2B and C). However, the Shannon diversity index and the inverse Simpson diversity index were significantly decreased in the UC+CKD group compared to that in the control or UC group (Figure S2D and E), suggesting that the bacterial diversity was lower in the UC + CKD group. Additionally, eGFR was significantly positively correlated with the Shannon index and the inverse Simpson index (Table S2). The principal component analysis showed an overlap to some extent among the three groups, indicating similar bacterial community structures (Figure S2F).

Among six bacterial phyla which accounted for over 97% of taxonomy in the gut microbiota of the study population, *Actinobacteria* exhibited the highest relative abundance in the UC + CKD group (Figure 1A). In addition, *Fusobacteria* was detected in some individuals with UC (Figure 1B). At the genus and species level, 24 genera and 23 species showed a relative abundance of more than 1% (Figure 1C and D).

**FIGURE 1:**
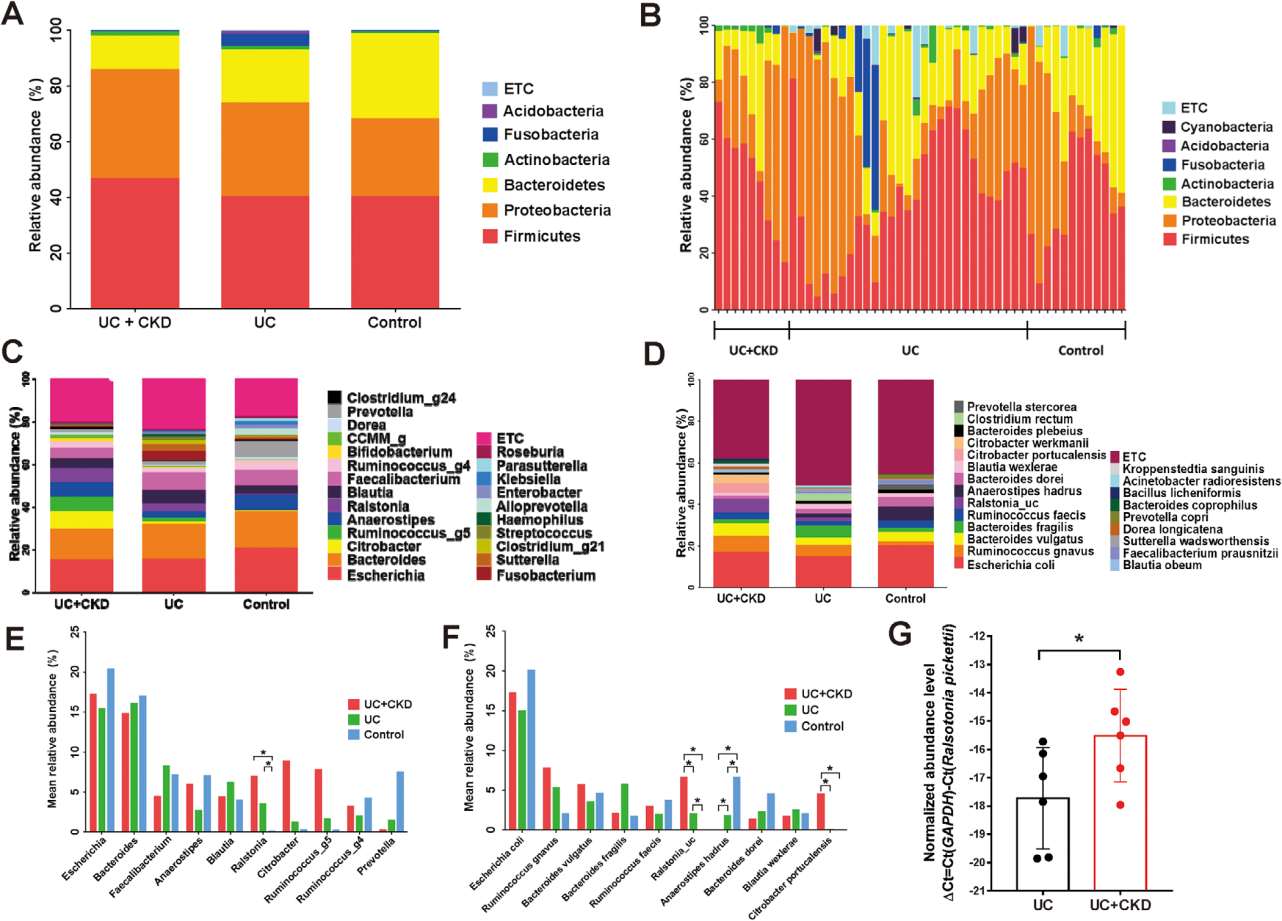
The taxonomic composition of the gut microbiota of the study population and comparison of the 10 most abundant genera and species in the UC + CKD, UC, and control groups. (A, B) The taxonomic composition of each group (A) and individual samples (B) at the phylum level. (C) The genera with greater than 1% relative abundance. (D) The species with greater than 1% relative abundance. (E) At the genus level, the abundance of *Ralstonia* was significantly higher in the UC + CKD and UC groups than that in the control group. (F) At the species level, the abundances of *Ralstonia_uc* and *Citrobacter portucalensis* were significantly higher in the UC + CKD group compared to those in the other groups, whereas the abundance of *Anaerostipes hadrus* was significantly higher in the control group compared to that in the other groups. (G) Real‐time PCR on all available RNA samples extracted from colon biopsy samples in the UC + CKD and UC groups validated the higher abundance of *Ralstonia pickettii* in the UC + CKD group than that of the UC group with statistical significance. *p < 0.05; CKD, chronic kidney disease; UC, ulcerative colitis

Of the 10 genera that were relatively abundant in the study population, only *Ralstonia* showed a higher relative abundance in the UC + CKD and UC groups compared to that in the control group (Figure 1E). Furthermore, *Ralstonia_uc* (unclassified *Ralstonia* species) showed the highest relative abundance in the UC + CKD group (Figure 1F). Additional real‐time PCR on colon biopsy samples of UC + CKD and UC patients using primers specific for *Ralstonia pickettii* indeed validated the presence of the microbe only in the UC + CKD group (Figure 1G).

When the correlation of relative abundance of microbes with eGFR in the UC + CKD group was examined, the relative abundance of seven genera including *Ralstonia* and four species including *Ralstonia_uc* showed a negative correlation with eGFR (Figure 2A; Figure S3A; Table S3). Furthermore, the relative abundances of *Ralstonia* and *Ralstonia_uc* showed a positive correlation with the serum uric acid level (Figure 2B; Figure S3B), whereas relative abundances of *Anaerostipes hadrus* and *Citrobacter portucalensis* did not correlate with eGFR and serum uric acid level (Figure S3C–F).

**FIGURE 2:**
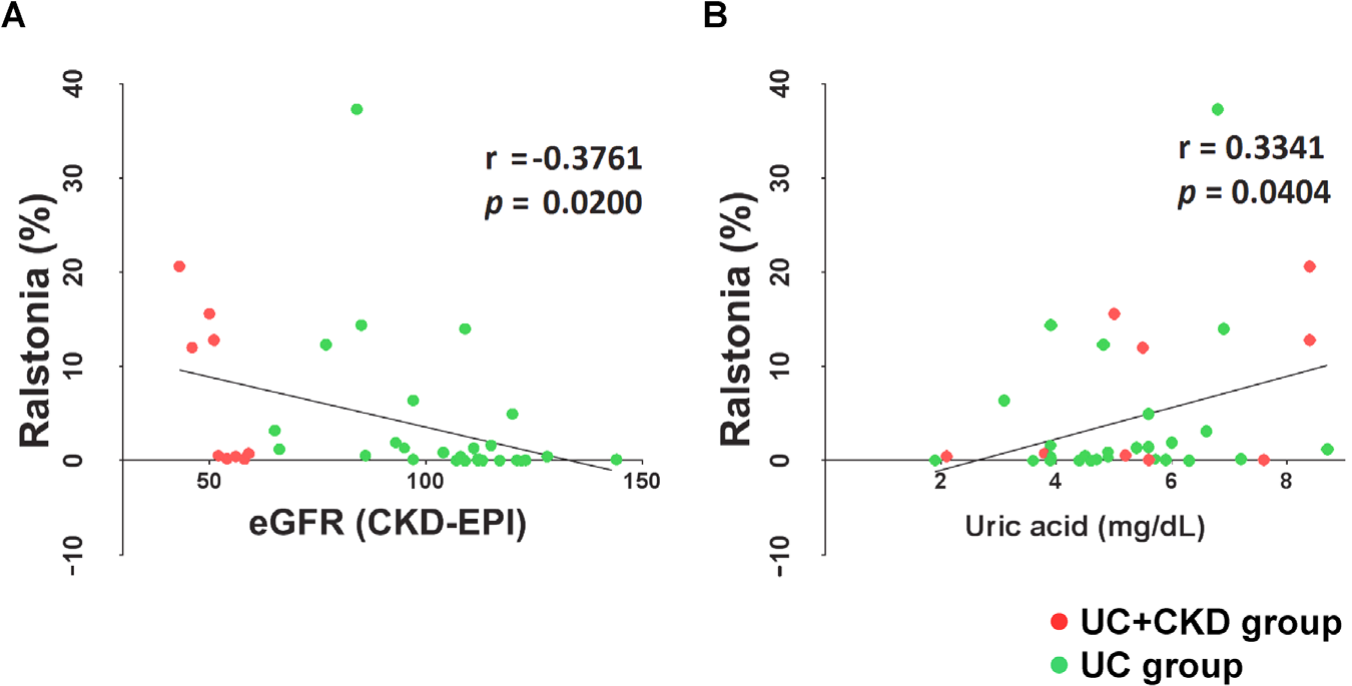
The correlation between the relative abundance of *Ralstonia* with renal function (indicated by eGFR) and serum uric acid level. (A, B) The relative abundance of *Ralstonia_uc* was negatively correlated with eGFR (CKD‐EPI) (A) and positively correlated with the serum uric acid level (B). Results of the UC + CKD group patients (red dots) and the UC group patients (green dots) were collectively analyzed

Among several species of the *Ralstonia* genus, we selected *R. pickettii* because it is a key member of the genus and implicated in human pathogenicity.^4^ Among various inflammatory markers associated with the UC patients, three pro‐inflammatory cytokine genes were chosen to examine the effect of *R. pickettii* treatment on Caco‐2 cells. The treatment of Caco‐2 cells with *R. pickettii* induced the upregulation of mRNA levels of these pro‐inflammatory cytokines (IL1β, IL6, and TNF‐α) by at least twofold compared to that of the untreated control samples (Figure 3A). This suggests that *R. pickettii* contributed to the inflammation seen in an UC *in vitro* assay resembling the intestinal conditions of UC patients.

**FIGURE 3:**
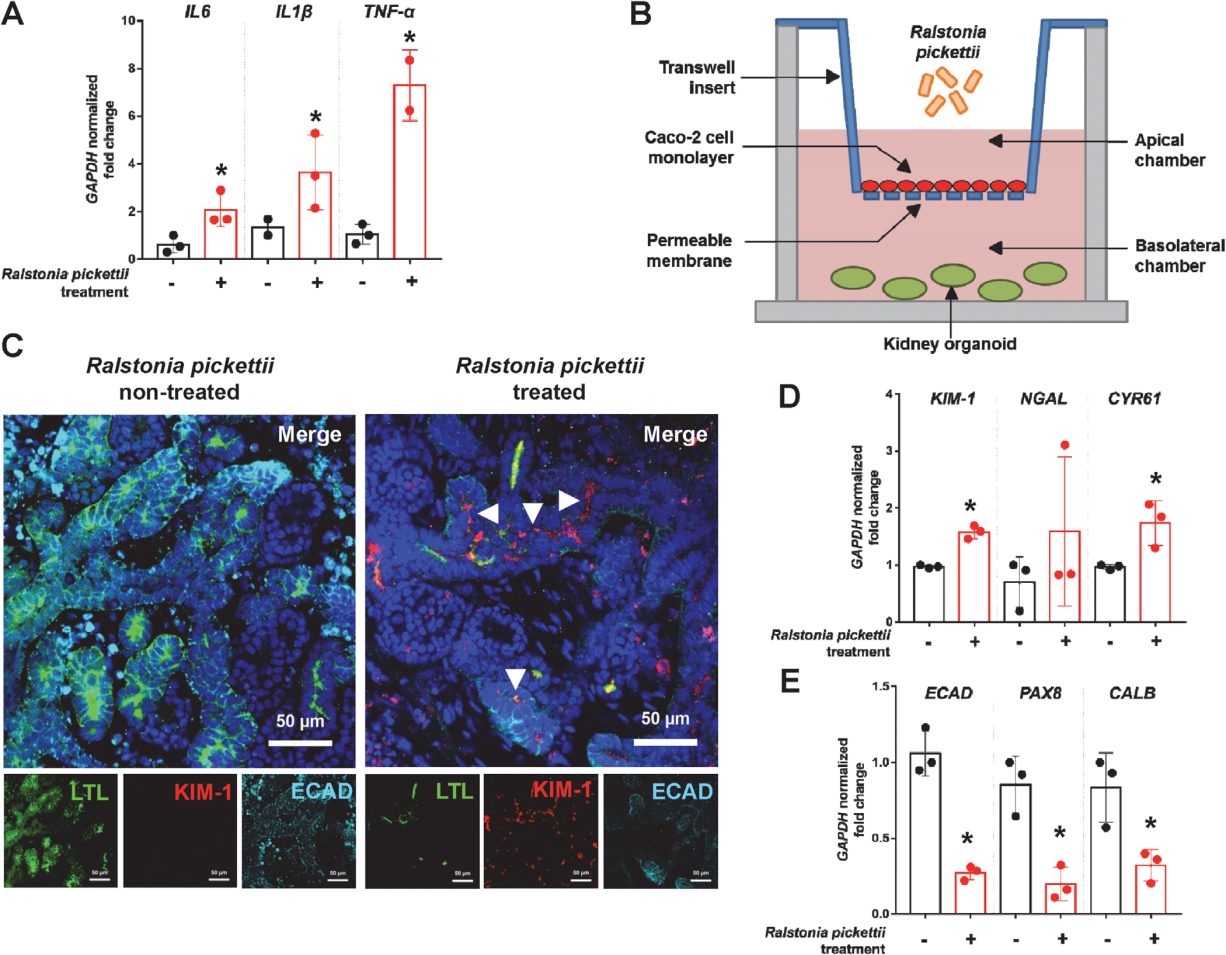
Effects of *Ralstonia pickettii* on Caco‐2 cells and kidney organoids. (A) mRNA expression of pro‐inflammatory cytokine genes in Caco‐2 cells treated with and without *Ralstonia pickettii*, reflecting the inflammation seen in ulcerative colitis. (B) Schematic diagram of the co‐culture experiment. (C) Immunofluorescence staining of LTL, KIM‐1, and E‐cadherin in kidney organoids treated with and without *Ralstonia pickettii*. (D, E) Expression of acute injury (D) and renal marker (E) genes in the control and *Ralstonia pickettii* treated kidney organoids

To model the gut–kidney axis, we utilized a co‐culture of Caco‐2 cells and kidney organoids in a transwell system; the kidney organoids were not directly exposed to *R. pickettii* (Figure 3B). Kidney organoids were generated from human‐induced pluripotent stem cells as described previously.^5^ *Ralstonia*‐treated kidney organoids presented markedly increased expression of *KIM‐1* compared to the untreated control samples (Figure 3C). On the contrary, mildly decreased expression of proximal tubule marker, lotus tetragonolobus lectin (LTL), and distal tubule marker, E‐cadherin, was observed in *R. pickettii* treated kidney organoids compared to the untreated control samples, indicating damage within the kidney tubules (Figure 3C). Furthermore, transcriptional activation of *KIM‐1* and *CYR61* in the *R. pickettii* treated organoids was statistically significant (Figure 3D), and the expression of the renal markers ECAD, PAX8, and CALB was significantly decreased to 0.26‐, 0.23‐, and 0.38‐fold, respectively (Figure 3E). It is interesting that not only the acute kidney injury markers but also the structural markers were affected by *Ralstonia* treatment, indicating that virulence factors such as endotoxins can provoke renal dysfunction through the gut–kidney axis.

In this study, we found that the abundance of *Ralstonia* was significantly higher in the UC + CKD group at both the genus and species levels. The relative abundance of *Ralstonia* was positively correlated with serum uric acid level and negatively correlated with eGFR. *Ralstonia* is an aerobic, gram‐negative, nonfermentable rod, and an opportunistic pathogen that causes infections in immunocompromised hosts. Intestinal *R. pickettii* is associated with the significant increase in endotoxin levels and worsened glucose intolerance in obesity.^6^ An increase in plasma endotoxin levels eventually results in systemic endotoxemia and chronic inflammation.^7^ Endotoxemia is a characteristic of CKD and is associated with the CKD stage and decreased survival.^8^

The limitations of this study include its cross‐sectional nature, relatively small number of patients, and relatively high variability in the microbial composition of each group. Additional studies with larger sample sizes might be necessary to confirm the findings of the present study. In addition, the results of this study will be strengthened by comparing the current data with those for the microbiome of patients with Crohn's disease and CKD, and CKD patients without IBD. Further studies are required regarding potential confounding factors such as contamination in the 16S rRNA microbiome studies.^9^ *In vitro* studies utilizing clinical isolates of *R. pickettii* from fecal culture samples might also validate our findings.

In conclusion, the present study demonstrates a change in the microbial community in patients with UC and decreased renal function. *R. pickettii* was negatively correlated with renal function in individuals with UC and associated with kidney‐specific disruptive features especially under the inflamed colon, suggesting that *R. pickettii* might play an important role in the pathogenesis of CKD in UC.

## CONFLICT OF INTEREST

All authors declare no conflict of interest.

## Supporting information



Supporting information
