# Supplementary material for: Microbiome analysis reveals that Ralstonia is responsible for decreased renal function in patients with ulcerative colitis
Source: Clin Transl Med. 2021 Mar 4;11(3):e322. doi: 10.1002/ctm2.322 (PMC7933010; doi:10.1002/ctm2.322)
Supplement: Supplementary file 1 — Supporting information [file CTM2-11-e322-s001.pdf]

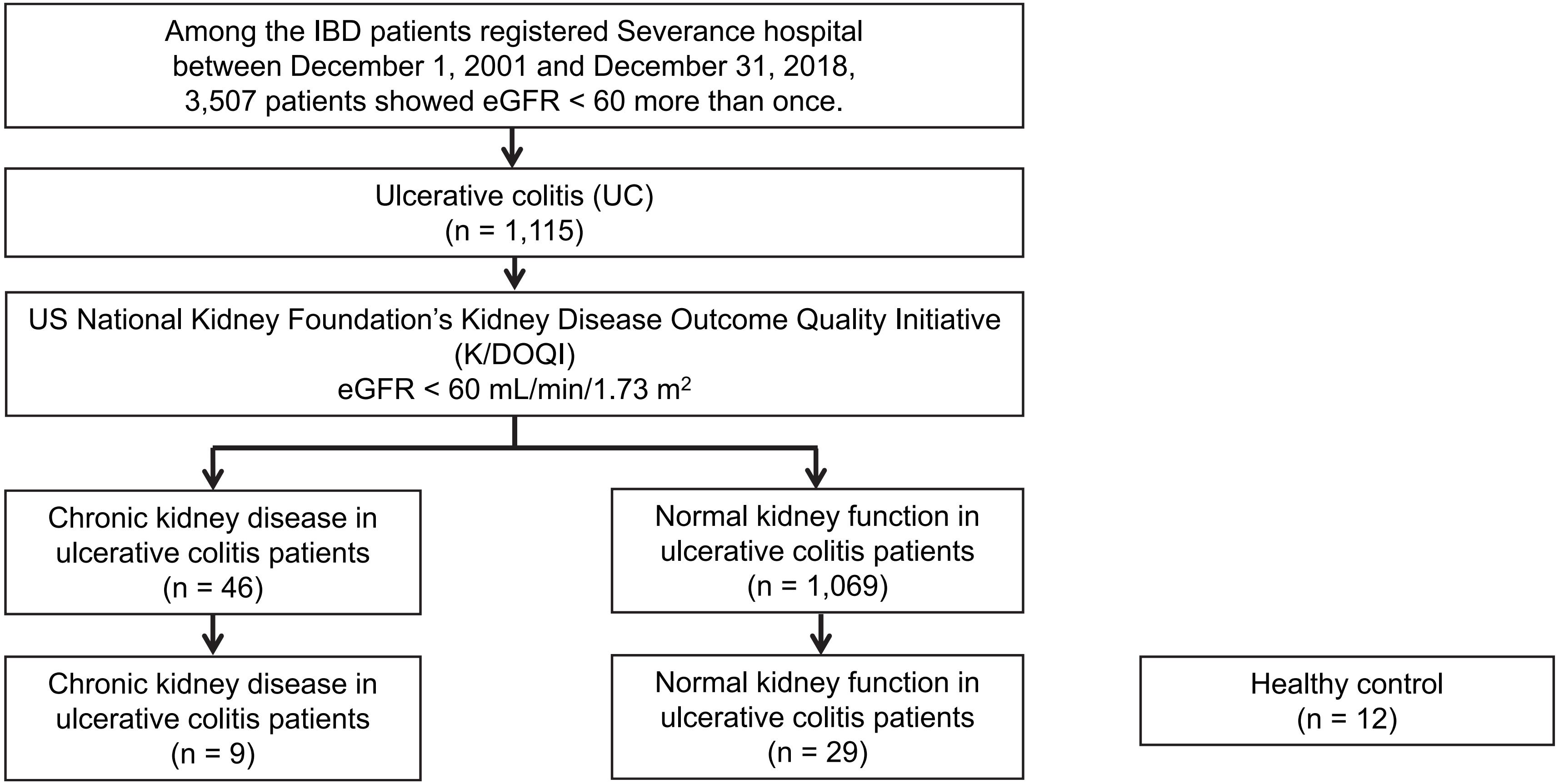

Figure S1. Flow chart of the study.

Of the 3,507 IBD patients who had an eGFR of less than 60 on at least one occasion, 1,115 individuals had ulcerative colitis (UC), of which 46 had chronic kidney disease (CKD) based on the K/DOQI definition. In total, nine of the 46 UC+CKD patients and 29 of the 1,069 UC patients with normal renal function were enrolled. The 16S rRNA sequencing of colonic tissue samples of these individuals was performed, and samples of 12 healthy individuals were included as a control group.

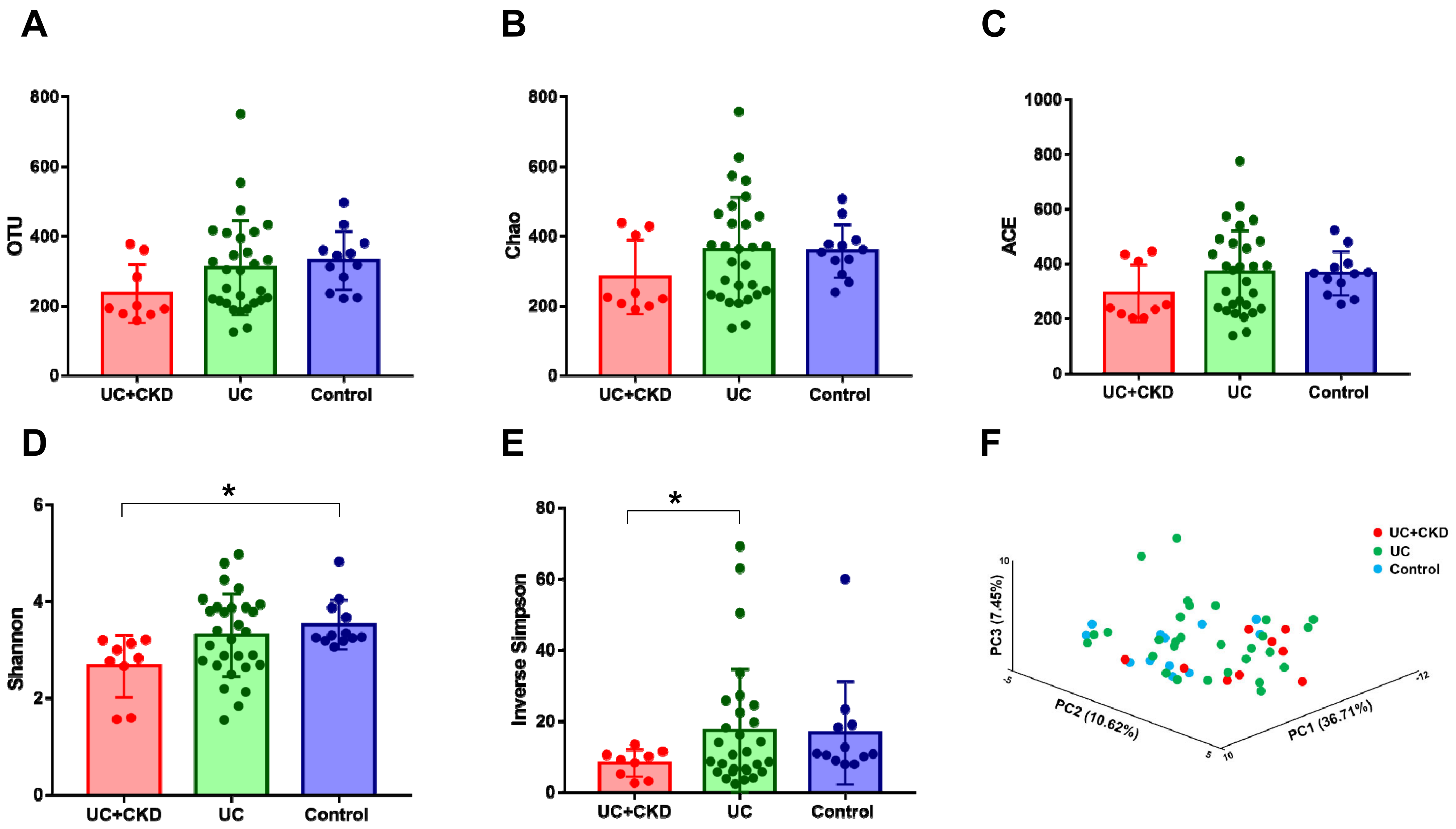

Figure S2. Characteristics of the gut microbial communities of patients in the UC+CKD, UC, and control groups.
(A) OTUs. (B, C) Community richness indices according to Chao1 (B) and ACE (C). (D, E) Alpha diversity indices. The Shannon diversity index of the UC+CKD group was significantly lower compared to that of the control group (D). The inverse Simpson index of the UC+CKD group was significantly lower compared to that of the UC group (E). (F) Principal component analysis (PCA) showed beta diversity among the UC+CKD group (red), UC group (green), and healthy (blue) individuals for the first three principal components. *P < 0.05; CKD, chronic kidney disease; UC, ulcerative colitis.

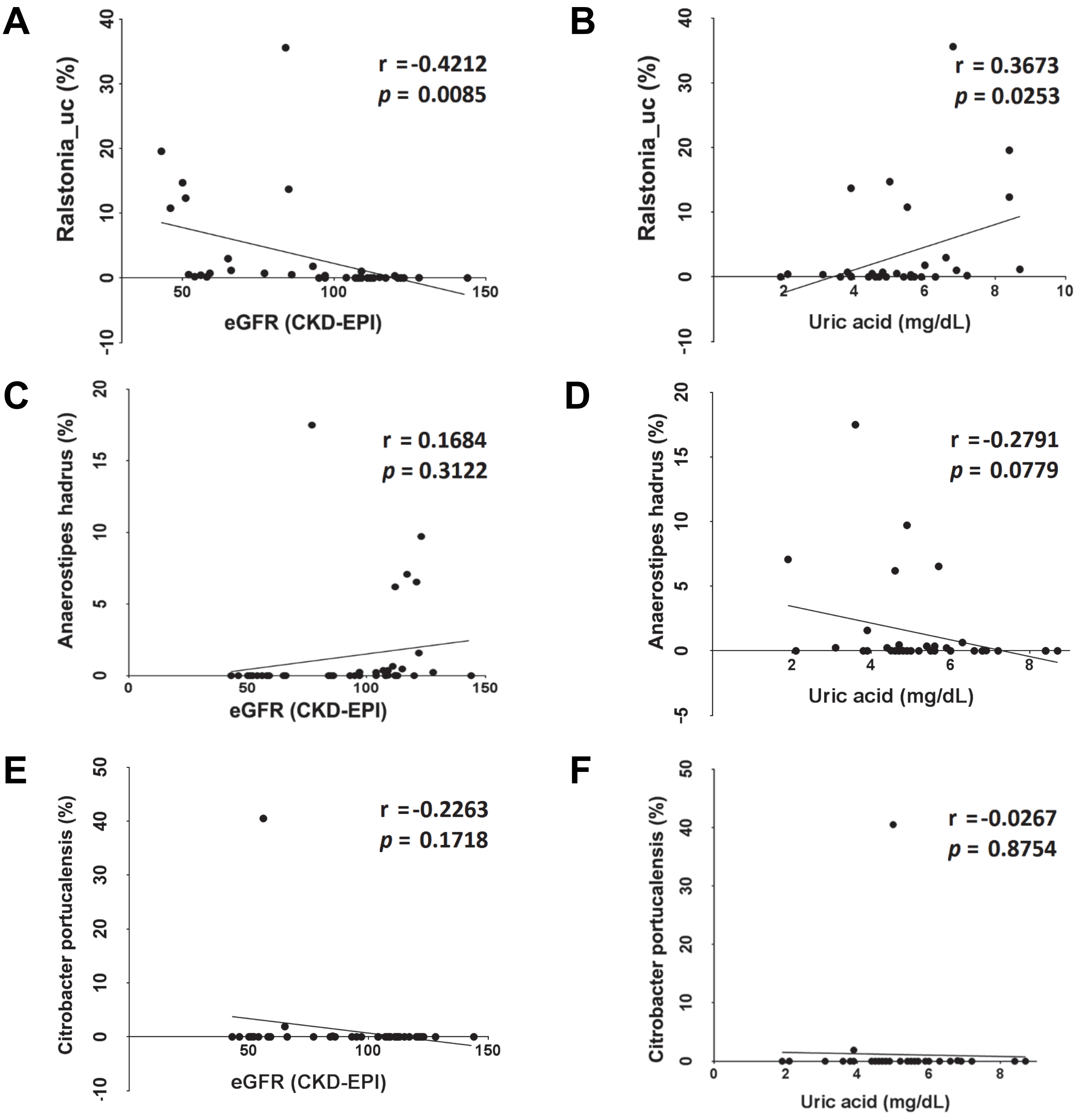

Figure S3. Correlation between the relative abundance of the gut microbes with renal function and serum uric acid.

(A, B) The relative abundance of *Ralstonia_uc* was negatively correlated with eGFR (CKD-EPI) (A) and positively correlated with the serum uric acid level (B).

(D-F) The relative abundances of *Anaerostipes hadrus* and *Citrobacter portucalensis* were not correlated with eGFR and serum uric acid.

Table S1. Patient demographics and baseline clinical characteristics

	UC + CKD (n = 9)	UC (n = 29)	Control (n = 12)
Age in year, mean ±SD	61.4 ± 9.0	48.8 ± 19.7	50.9 ± 14.4
Sex			
Male	6 (66.7)	17 (58.6)	6 (50.0)
Female	3 (33.3)	12 (41.4)	6 (50.0)
Hypertension, n (%)	3 (33.3)	5 (17.2)	1 (8.3)
DM, n (%)	1 (11.1)	3 (10.3)	1 (8.3)
Smoking (n, %)	3 (33.3)	10 (34.5)	4 (33.3)
BUN (mg/dL)[a]	19.8 ± 6.1	12.5 ± 3.8	
Creatinine (mg/dL)[a]	1.4 ± 0.2	0.7 ± 0.2	
Uric acid (mg/dL)	5.6 ± 2.1	5.0 ± 1.3	
Duration of UC in year, mean ± SD	6.8 ± 4.7	5.6 ± 4.9	
Mayo Clinic Score			
≤2 and no subscore > 1	0	3 (10.3)	
3-5	7 (77.8)	9 (31.0)	
6-10	2 (22.2)	17 (58.6)	
11-12	0	0	
Montreal			
E1 (Ulcerative proctitis)	3 (33.3)	9 (31.0)	
E2 (Left sided ulcerative colitis)	3 (33.3)	10 (34.5)	
E3 (Extensive ulcerative colitis)	3 (33.3)	10 (34.5)	
Medication			
Mesalamine (asacol/pentasa)	4 (44.4)	12 (46.2)	
Mesalamine suppo. (asacol/pentasa)	4 (44.4)	15 (57.7)	
Balsalazide (colazal)	3 (33.3)	9 (34.6)	
Sulfasalazine (salazopyrin)	1 (11.1)	3 (11.5)	
AZA	2 (22.2)	5 (19.2)	
Anti-TNFα biologics	1 (11.1)	4 (15.3)	

Data are presented as n (%). AZA: azathioprine; BUN: blood urea nitrogen; CKD: chronic kidney disease; DM: diabetes mellitus type 2; SD: standard deviation; UC: ulcerative colitis

[a]: p value < 0.05

Table S2. Multivariate linear regression analyses of log-transformed diversity measures

Parameter	Chao		Ace		Shannon index		Inverse Simpson	
	Beta (95% CI)	*p* value	Beta (95% CI)	*p* value	Beta (95% CI)	*p* value	Beta (95% CI)	*p* value
eGFR	0.004 (-0.003 - 0.011)	0.270	0.004 (-0.003 - 0.011)	0.308	0.006 (0.001 - 0.010)	0.013	0.018 (0.005 - 0.031)	0.009
Mayo Clinic score	-0.047 (-0.112 - 0.018)	0.151	-0.048 (-0.112 - 0.017)	0.140	-0.009 (-0.051 - 0.033)	0.657	-0.008 (-0.129 - 0.112)	0.889
Duration of UC, year	0.000 (-0.003 - 0.002)	0.723	0.000 (-0.003 - 0.002)	0.715	-0.001 (-0.003 - 0.001)	0.251	-0.003 (-0.008 - 0.003)	0.300
Age, year	0.007 (-0.005 - 0.020)	0.250	0.007 (-0.005 - 0.020)	0.243	0.007 (-0.001 - 0.015)	0.097	0.026 (0.003 - 0.050)	0.026
Hypertension	-0.043 (-0.423 - 0.337)	0.820	-0.051 (-0.426 - 0.323)	0.782	-0.220 (-0.465 - 0.025)	0.076	-0.645 (-1.349 - 0.058)	0.071

Table S3. Negative correlation between species with relative abundance and GFR (CKD-EPI)

Parameter	r (95% CI)	R^2	*P* value
Species with relative abundance greater than 1% in UC+CKD group			
*Ralstonia*_uc	-0.4212 (-0.6529 - -0.1173)	0.1774	0.0085
Anaerostipes hadrus group	-0.5361 (-0.7306 - -0.2612)	0.2874	0.0005
Faecalibacterium prausnitzii group	-0.4050 (-0.6416 - -0.0980)	0.164	0.0117
Species with relative abundance less than 1% in UC+CKD group			
Cutibacterium acnes group	-0.4822 (-0.6948 - -0.1922)	0.2325	0.0022
Collinsella aerofaciens group	-0.4489 (-0.6721 - -0.1509)	0.2015	0.0047
Coprococcus comes group	-0.4026 (-0.6399 - -0.0952)	0.1621	0.0122
Staphylococcus aureus group	-0.4333 (-0.6614 - -0.1319)	0.1878	0.0066
Lactobacillus rogosae group	-0.3659 (-0.6138 - -0.0523)	0.1338	0.0239
Pseudomonas fulva group	-0.3834 (-0.6263 - -0.0726)	0.147	0.0175
Eubacterium eligens group	-0.4776 (-0.6917 - -0.1864)	0.2281	0.0024
Bacteroides xylanisolvens group	-0.5379 (-0.7318 - -0.2635)	0.2893	0.0005
Blautia hansenii group	-0.3416 (-0.5962 - -0.0246)	0.1167	0.0358
Paraburkholderia insulsa	-0.4844 (-0.6962 - -0.1948)	0.2346	0.0021
Ralstonia solanacearu group	-0.4529 (-0.6749 - -0.1558)	0.2052	0.0043
Paraburkholderia fungorum	-0.3795 (-0.6235 - -0.0681)	0.144	0.0188
Cupriavidus metallidurans	-0.3876 (-0.6293 - -0.0775)	0.1502	0.0162
Ralstonia pickettii group	-0.4443 (-0.6689 - -0.1452)	0.1974	0.0052
Agathobaculum butyriciproducens group	-0.5747 (-0.7555 - -0.3124)	0.3302	0.0002

Supplementary Table S4. Primers for real-time PCR performed in this study

Gene	Species	Forward sequence	Reverse sequence
KIM-1	Human	CGTCCACCGCAAATGCTT	TCTGCGCAAGTTAGGTTTTGTC
NGAL	Human	GAAGACAAAGACCCGCAAAAG	CTGGCAACCTGGAACAAAAG
CYR61	Human	GAGTGGGTCTGTGACGAGGAT	GGTTGTATAGGATGCGAGGCT
ECAD	Human	CACCGATGGTGAGGGTACACAG	GGCTTCAGGAATACATGGACAAAGA
PAX8	Human	CTGAGGGCGTCTGTGACAATG	TGAATGGTTGCTGCACTTTGG
CALB	Human	GATACTGACCACAGTGGCTTCATAG	GCCATCTCAGTTAATTCCAGCTTC
IL1β	Human	CCACAGACCTTCCAGGAGAATG	GTGCAGTTCAGTGATCGTACAGG
IL6	Human	AGACAGCCACTCACCTCTTCAG	TTCTGCCAGTGCCTCTTTGCTG
TNF-α	Human	CTCTTCTGCCTGCTGCACTTTG	ATGGGCTACAGGCTTGTCACTC
GAPDH	Human	CCAGCCGAGCCACATCGCTC	ATGAGCCCCAGCCTTCTCCAT

SUPPLEMENTARY MATERIALS AND METHODS

1. Study population

This study was approved by the institutional review board of the Severance Hospital, Yonsei University Health System (IRB#4-2019-0030), Seoul, Korea. All research was performed in accordance with the relevant regulations, and written informed consent was obtained from all subjects prior to their enrollment. Among the 3,507 patients with IBD who visited Severance Hospital from December 1, 2000 to December 31, 2018 and showed estimated glomerular filtration rate (eGFR) values less than 60 mL/min/1.73 m^2 more than once, 1,115 patients had UC. Forty-six of the 1,115 patients developed CKD that persisted for a minimum of three months. Nine of the 47 patients with both UC and CKD, and 29 of the 1,069 patients with UC with normal renal function were recruited in this study. Twelve healthy individuals who had no surgical or comorbidity history and had normal colonoscopy results were also recruited as controls (Figure S1).

The eGFR for eligibility was determined using the CKD-EPI equation[1] and CKD is defined as per the US National Kidney Foundation's Kidney Disease Outcome Quality Initiative (K/DOQI) clinical practice guidelines.[2] The individuals with intercurrent illnesses, acute or chronic infections, and a history of antibiotic therapy over the prior three months were excluded. All mucosal samples were obtained from the normal ileocecal area via colonoscopic biopsy. The DNA of microbial flora contained in the tissue samples of the ileocecal area was isolated using the FastDNA® SPIN Kit for Soil (MP Biomedicals, USA).

2. Sequencing and PCR amplification

The V3-V4 region of the bacterial 16S rRNA gene was amplified using a specific primer

with adaptors and barcodes. The PCR amplicons were pooled and concentrated to a volume of 40 μL. AMPure beads XP (Beckman Coulter, Indianapolis, USA) were used for washing and size selection of each PCR product, and each purified PCR product was quantified using a Quat-iT PicoGreen dsDNA assay kit (Molecular Probes, USA). The quality and size of the products were assessed on an Agilent 2100 Bioanalyzer (Agilent Technology) using an Agilent DNA 7500 kit, and the libraries were quantified using a fluorometric quantification method that uses dsDNA binding dyes or qPCR. For qPCR, a KAPA library quantification kit and KAPA standard kit (Kapa Biosystems) were utilized. The library was sequenced on an Illumina MiSeq platform at Chunlab Inc. (Seoul, Korea).

3. Microbiome data analysis

The low quality (<Q25) reads were filtered out using Trimmomatic 0.32. After the QC pass, the paired-end sequence data were merged using PANDAseq. The primers were trimmed at a similarity cut-off of 0.8, and non-specific amplicons that did not encode 16S rRNA were detected using HMMER's hmmsearch program with a 16S rRNA profile. Each read was assigned to samples based on its unique barcode and merged using CLcommunity™ (version 3.4.2 http://data.chunlab.com/software). In addition to the 16S rRNA gene sequences that were previously maintained at the EzTaxon database (http://www.eztaxon.org/), type or representative 16S rRNA gene sequences were collected from Candidatus taxa and species with non-validly published names from public domain databases, including PubMed and GenBank. Moreover, the 16S rRNA gene sequences that can be found on assembled contigs of genome and metagenome sequencing projects were extracted using the rRNASelector program (http://sw.ezbiocloud.net/rrnaselector). To compare the operational taxonomic units

(OTUs) between samples, shared OTUs were obtained through the XOR analysis in CLcommunity™. Richness and diversity of the samples were analyzed using the abundance-based coverage estimators (ACEs), Chao1, Shannon, and Simpson indexes. In addition, the Simpson and Shannon diversity indexes at 3% distance were calculated in CLcommunity™. The alpha and beta diversities were displayed using the R package (version 3.6.1). The taxonomic name suffix "_uc" stands for unclassified and is a collection of sequences that can be considered new species because there are no similar sequences at the species level in the database. For example, if a sequence was identified as belonging to the genus *Ralstonia* but not identified at the species level, it was named as *Ralstonia_uc*.

4. Real-time PCR for *Ralstonia pickettii* from biopsy samples

For validation of presence of Ralstonia pickettii in biopsy samples, real-time PCR on RNA samples extracted from the all available conoloscopy biopsy samples using RiboEx (GeneAll Biotechnology) and reverse-transcribed with an iScript cDNA Synthesis Kit (Bio-Rad) was performed. The samples were assayed with SYBR Green ready master mix and the appropriately designed primers validated in the previous study were used (forward primer sequence: ATGATCTAGCTTGCTAGATT GAT; reverse primer sequence: ACTGATCGTCGCCTTGGTG).[3] Real-time PCR was performed with the CFX96 Real-Time PCR Detection System (Bio-Rad, USA). The relative RNA expression levels were calculated via a comparative threshold cycle (Ct) method and glyceraldehyde 3-phosphate dehydrogenase (*GAPDH*) was used as a control.

4. *Ralstonia pickettii* culture conditions

The bacterial strain *R. pickettii* (ATCC 27511) was cultured in Difco nutrient broth (#234000, BD company, USA) and agar (#213000, BD company) at 30°C according to the ATCC protocol. The identity of the bacterial isolate was confirmed using matrix-assisted laser desorption ionization time-of-flight mass spectrometry (MALDI-TOF MS, Bruker Daltonics) as *R. pickettii*, according to the manufacturer's protocol, with a score of 2.006.

5. Culture of Caco-2 cells, kidney organoids, and *R. pickettii*

Caco-2 cells (ATCC) were cultured in Dulbecco's modified essential medium (DMEM) (Thermo Fisher) supplemented with 20% FBS, penicillin (50 IU/mL)/streptomycin (50 μg/mL), and 2 mM L-glutamine (Thermo Fisher). The Caco-2 cells were differentiated and maintained for 2-3 weeks in a transwell system (#3460, Corning, USA), and the medium was changed every 2 days. To evaluate the mRNA expression patterns of inflammatory genes, including *IL-1β*, *IL-6*, and *TNF-α*, confluent Caco-2 cells were treated with 1 mL of *R. pickettii* broth (7.0 x 10^8 CFU/mL) in 12-well plates for 2 hours.

Kidney organoids were generated from human induced pluripotent stem cells (hiPSCs) (#WISCi004-B, WiCell) and maintained in mTeSR1 (#85850, STEMCELL Technologies), as described previously.[4,5] Briefly, hiPSCs were induced into posterior intermediate mesoderm lineage cells upon sequential treatment with high concentration of CHIR99021 (SML1046, Sigma), human noggin (#120-10C, PeproTech), and activin A (#338-AC, R&D systems). On day 9, the induction of nephron progenitor cells was confirmed by the presence of SIX2-positive cell populations. The cells were treated with fibroblast growth factor-9 (273-F9, R&D systems) from day 7 to day 14. Additionally, pulsatile treatment with CHIR99021 was performed from day 9 to day 11, and the nephron progenitor cell populations were

differentiated to pre-tubular aggregates. On day 21, the differentiation was terminated, and the renal vesicle structure and kidney organoids were stained for lotus tetragonolobus lectin (LTL) and E-cadherin (ECAD) to verify intact formation of kidney tubules structures.

When the kidney organoids were fully differentiated, a transwell containing confluent Caco-2 cells was inserted over each organoid. The transepithelial electrical resistance (>1200 Ω/cm^2) was measured for the tight junctions in Caco-2 monolayers using an EVOM2 epithelial volt-ohmmeter (World Precision Instruments, USA). The Caco-2 cells with appropriate transepithelial resistance were treated with *R. pickettii* (7.0 x 10^8 CFU/mL) to investigate the effects of *R. pickettii* bypassing the intestinal epithelial layer on the kidney organoids. Locus-specific expression of kidney and injury markers [LTL, ECAD, and kidney injury molecule-1 (KIM-1)] was evaluated via immunofluorescence. Further, mRNA expression levels of kidney injury markers [cysteine-rich angiogenic inducer 61 (CYR61), KIM-1, neutrophil gelatinase-associated lipocalin (NGAL)] and renal markers [calbindin (CALB), ECAD, PAX8] were compared between *R. pickettii*-treated and untreated kidney organoids.

6. Immunofluorescence and real-time PCR

For immunofluorescence, the kidney organoids were fixed by submersion in 4% formaldehyde at 4 °C overnight. The fixed organoids were washed three times in phosphate buffered saline (PBS) and incubated in blocking buffer containing 10% donkey serum and 0.3% triton X-100 in PBS for 1 hour at room temperature. For staining, 1:100 and 1:1000 dilutions of primary and fluorophore-tagged secondary antibodies, respectively, were used. Alexa 568-, and Alexa 647-conjugated secondary antibodies and 4′,6-diamidino-2-phenylindole dihydrochloride were obtained from

Invitrogen. Confocal images were obtained with a Carl Zeiss LSM780 instrument and ZEN software was used for image processing. Anti-LTL (#FL-1321, Vector Labs) anti-KIM-1 (#AF1750, R&D), and anti-E-cadherin (#610181, BD) antibodies were purchased from commercial sources.

For real-time PCR, RNA samples were isolated from the Caco-2 cells and kidney organoids with RiboEx (GeneAll Biotechnology) and reverse-transcribed with an iScript cDNA Synthesis Kit (Bio-Rad). The samples were assayed with SYBR Green ready master mix and ROX reference dye (Takara Bio); primers are listed in Table S4. Real-time PCR was performed with the CFX96 Real-Time PCR Detection System (Bio-Rad, USA). The relative RNA expression levels were calculated via a comparative threshold cycle (Ct) method and glyceraldehyde 3-phosphate dehydrogenase (*GAPDH*) was used as a control.

7. Statistical analyses

Statistical analysis was performed using the R package and the Statistical Package for Social Sciences version 22.0 (SPSS Inc., Chicago, IL, United States). The Kolmogorov-Smirnov test was used to test whether the data were distributed normally. For normally distributed data, a one-way analysis of variance (ANOVA) with Tukey multiple comparison test was used. If data were not normally distributed, non-parametric Kruskal-Wallis one-way ANOVA followed by Dunn's post hoc test and the Mann-Whitney test were used for independent samples. The Wilcoxon Signed Rank test was used when the samples were dependent. The Benjamini-Hochberg false discovery rate (FDR) was used to adjust multiple tests, as appropriate. Analysis of similarities (ANOSIM) was used to test for differences in total richness between the sample groups.[6] The Spearman correlation was used to analyze the correlation

between intestinal bacterial abundance and eGFR (CKD-EPI). $p < 0.05$ was considered statistically significant. All *in vitro* experiments were performed independently at least three times for statistical analyses.

REFERENCES

1. Levey AS, Stevens LA, Schmid CH, et al. A new equation to estimate glomerular filtration rate. *Ann Intern Med.* 2009;150(9):604-612.
2. Inker LA, Astor BC, Fox CH, et al. KDOQI US commentary on the 2012 KDIGO clinical practice guideline for the evaluation and management of CKD. *Am J Kidney Dis.* 2014;63(5):713-735.
3. Udayappan SD, Kovatcheva-Datchary P, Bakker GJ, et al. Intestinal Ralstonia pickettii augments glucose intolerance in obesity. *PLoS One.* 2017;12(11):e0181693.
4. Morizane R, Bonventre JV. Generation of nephron progenitor cells and kidney organoids from human pluripotent stem cells. *Nat Protoc.* 2017;12(1):195-207.
5. Morizane R, Lam AQ, Freedman BS, Kishi S, Valerius MT, Bonventre JV. Nephron organoids derived from human pluripotent stem cells model kidney development and injury. *Nat Biotechnol.* 2015;33(11):1193-1200.
6. CLARKE KR. Non-parametric multivariate analyses of changes in community structure. *Australian Journal of Ecology.* 1993;18(1):117-143.
